# Exploring the potential mechanism of the Chinese medicine Duhuo on rheumatoid arthritis based on network pharmacology

**DOI:** 10.1097/MD.0000000000045768

**Published:** 2026-01-02

**Authors:** Shouyu Cao, Hongbo Li, Hui Lv, Haorui Shi, Zilong Zhang

**Affiliations:** a College of Traditional Chinese Medicine, Changchun University of Traditional Chinese Medicine, Changchun, Jilin, China; b Department of Rheumatology and Immunology, The Third Affiliated Hospital of Changchun University of Traditional Chinese Medicine, Changchun, Jilin, China.

**Keywords:** Duhuo, network pharmacology, rheumatoid arthritis, the signaling pathway

## Abstract

According to current research, the main components of *Duhuo*, such as volatile oils and coumarins, possess anti-inflammatory and antitumor properties. However, the precise mechanisms underlying these effects remain unclear. This study aimed to investigate the potential mechanisms of *Duhuo* in the treatment of rheumatoid arthritis (RA) using network pharmacology. The TCMSP, GeneCards, and DisGeNET databases were used to identify the active ingredients and target genes of *Duhuo*, as well as the RA-related genes. Common targets were determined using a Venn diagram. Cytoscape software was employed to construct protein–protein interaction (PPI) networks, and the core targets were identified and analyzed. Gene ontology (GO) and Kyoto Encyclopedia of Genes and Genomes (KEGG) enrichment analyses were performed using the DAVID database to visualize functional pathways. A total of nine active compounds in *Duhuo*, 274 potential target genes, and 96 overlapping genes associated with both *Duhuo* and RA were identified. PPI network analysis revealed SRC, EGFR, CASP3, PPARG, and PTGS2 as the core targets of *Duhuo* in RA treatment. Enrichment results indicated involvement in cancer signaling, the MAPK pathway, PI3K-Akt signaling, and the prolactin signaling pathway. These findings suggest that *Duhuo* exerts anti-RA effects through multiple components, targets, and signaling pathways.

## 1. Introduction

Rheumatoid arthritis (RA) is a chronic autoimmune disorder associated with a high global disability rate. According to the World Health Organization (WHO), the global incidence of RA is estimated to be approximately 0.5 to 1.0%.^[1]^ RA is characterized by persistent synovial inflammation, pannus formation, and the progressive destruction of cartilage and bone tissue.^[2]^ It results not only in joint deformities and functional impairment but also affects multiple organ systems – including the cardiovascular, respiratory, and nervous systems – thereby substantially decreasing quality of life and increasing the societal and healthcare burden.^[3–5]^ Recent studies have revealed that RA pathogenesis involves a complex regulatory network, including T cell-mediated immune dysregulation, cascade activation of pro-inflammatory cytokines (e.g., TNF-α, IL-6, IL-17), abnormal proliferation of synovial fibroblasts, and osteoclast activation.^[6–9]^ Furthermore, interactions among HLA-DRB1 allele polymorphisms, gut microbiota imbalances, and environmental factors (such as smoking) contribute to the exacerbation of disease progression.^[10–12]^ Conventional pharmacological treatments for RA primarily include nonsteroidal anti-inflammatory drugs (NSAIDs), immunosuppressants, glucocorticoids, and a range of biologic agents. However, these therapies primarily alleviate symptoms and are often associated with adverse effects. Therefore, identifying safe and effective alternative therapies for RA remains an urgent clinical priority.

In recent years, traditional Chinese medicine (TCM) has played a significant role in the prevention and treatment of RA. According to TCM theory, RA primarily results from external pathogenic factors invading the limbs, obstructing the flow of qi and blood in the meridians, and ultimately causing persistent joint pain. Clinically, herbal medicines that dispel wind and dampness and promote collateral circulation to relieve pain are commonly employed. These treatments can significantly alleviate symptoms, enhance quality of life, and slow disease progression. For example, *Duhuo* is described in the *Compendium of Materia Medica*^[13]^ as “an herb that nourishes the blood and dispels wind, dampness, and cold.”

With the growing development of TCM, its therapeutic efficacy has gained increasing recognition, and elucidating its pharmacological mechanisms and potential medicinal value has become a critical issue requiring urgent attention. Network pharmacology, as a powerful tool for investigating drug–disease associations, enables precise analysis of drug–target interactions and provides deeper insights into underlying mechanisms of action. In this study, network pharmacology was employed to investigate the TCM *Duhuo*, identify its key components, therapeutic targets, and associated pathways, and to predict its potential mechanisms of action.

## 2. Methods and materials

### 2.1. Identification of key components and associated target genes of *Duhuo*

A search for “*Duhuo*” was performed in the TCM Systems Pharmacology Database and Analysis Platform (TCMSP) (https://www.tcmsp-e.com/load_intro.php?id=43), ^[14]^ and its primary components were screened based on criteria of oral bioavailability (OB) ≥ 30% and drug-likeness ≥ 0.18. The PubChem database (https://pubchem.ncbi.nlm.nih.gov/) was used to retrieve the SMILES structural formulas of the active compounds, which were subsequently submitted to the SwissTargetPrediction database (http://swisstargetprediction.ch/) for target prediction.^[15]^ As a result, the potential molecular targets of the active components of *Duhuo* were identified.

### 2.2. Collection of RA-related target genes

The term “rheumatoid arthritis” was searched in both the GeneCards (https://www.genecards.org/)^[16]^ and DisGeNET (https://www.disgenet.org/)^[17]^ databases to identify associated genes, which were then downloaded and saved for further analysis. Due to the high number of entries in the GeneCards database, genes with relevance scores below the median were excluded. The remaining genes were merged with those from the DisGeNET database, and duplicates were removed to obtain a final list of RA-associated target genes.

### 2.3. Identification of overlapping genes between *Duhuo* targets and ra-associated genes

The Bioinformatics platform (http://www.bioinformatics.com.cn/) was used to input the RA-associated genes and the *Duhuo*-related target genes, and a Venn diagram was generated to identify the overlapping genes shared by RA and *Duhuo*.

### 2.4. Identification of overlapping genes between *Duhuo* targets and RA-associated genes

The bioinformatics platform (http://www.bioinformatics.com.cn/) was used to input the RA-associated genes and *Duhuo*-related target genes, and a Venn diagram was generated to identify the intersecting targets.

### 2.5. Construction of drug–component–target and protein–protein interaction (PPI) networks

The key target genes of *Duhuo* and the intersecting genes with RA were imported into Cytoscape 3.10.0^[18]^ to construct a visualized network illustrating the relationships among the drug, active compounds, target genes, and disease. The overlapping genes were also imported into the STRING database (https://cn.string-db.org/)^[19]^ for PPI analysis. Homo sapiens was selected as the target species, with a minimum interaction confidence threshold of 0.4. Unconnected or irrelevant proteins were excluded to construct the final PPI network for *Duhuo* in RA treatment.

### 2.6. Gene ontology (GO) enrichment and Kyoto Encyclopedia of Genes and Genomes (KEGG) pathway analysis

The overlapping genes between *Duhuo* and RA were uploaded to the DAVID database (https://davidbioinformatics.nih.gov/), ^[20]^ with Homo sapiens selected as the reference species. Gene ontology (GO) enrichment and Kyoto Encyclopedia of Genes and Genomes (KEGG) pathway analyses were conducted using the Bioinformatics platform to explore the potential mechanisms of *Duhuo* in the treatment of RA.

## 3. Results

### 3.1. Key active components of *Duhuo*

A total of 99 chemical constituents of *Duhuo* were retrieved from the TCMSP database using “Duhuo” as the search term. Based on screening thresholds of OB ≥ 30% and drug-likeness ≥ 0.18, and in combination with relevant literature, nine major active components were identified. The target proteins of these active compounds were predicted using the PubChem and SwissTargetPrediction platforms, and subsequently converted into corresponding gene names. After removing duplicate entries, 274 target genes were identified for the potential active components of *Duhuo*, including F2R, PTGS2, PDE3A, PTPN1, and TOP2A. Detailed information is presented in Table 1.

**Table 1:** Primary active components and associated metrics of *Duhuo*.

Mol ID	Molecule name	OB (%)	DL
MOL000358	Beta-sitosterol	36.91	0.75
MOL001941	Ammidin	34.55	0.22
MOL001942	Isoimperatorin	45.46	0.23
MOL003608	O-acetylcolumbianetin	60.04	0.26
MOL004777	Angelol D	34.85	0.34
MOL004778	[(1R,2R)-2,3-dihydroxy-1-(7-methoxy-2-oxochromen-6-yl)-3-methylbutyl] (Z)-2-methylbut-2-enoate	46.03	0.34
MOL004780	Angelicone	30.99	0.19
MOL004782	[(1R,2R)-2,3-dihydroxy-1-(7-methoxy-2-oxochromen-6-yl)-3-methylbutyl] 3-methylbutanoate	45.19	0.34
MOL004792	Nodakenin	57.12	0.69

DL = drug-likeness, OB = oral bioavailability.

### 3.2. Overlapping genes between drug targets and RA-associated genes

**Figure 1.**
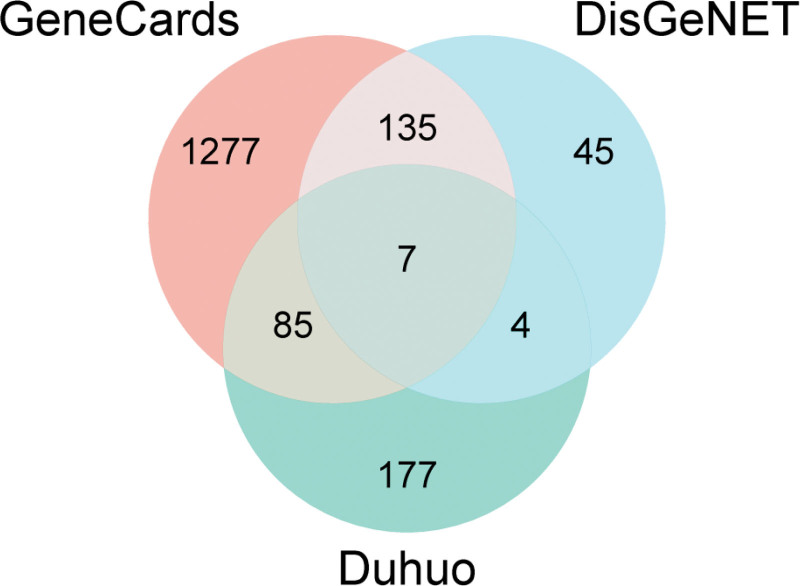
Venn diagram. There were 1504 target genes from the GeneCards database and 190 target genes from the DisGeNET database. 273 genes derived from Duhuo were intersected with RA genes, among which 96 genes overlapped. The proportion of overlapping genes was 35%, indicating that Duhuo has potential effects in treating RA, primarily comprising F2R, PTGS2, PDE3A, HSP90AB1, CYP1A2, and others. RA = rheumatoid arthritis.

Using “rheumatoid arthritis” as the keyword, gene searches were conducted in the GeneCards and DisGeNET databases. A total of 1504 RA-related genes were identified from GeneCards, while 190 genes were retrieved from DisGeNET. Upon merging the results, 142 duplicate genes were identified. After deduplication, a total of 1552 unique RA-related genes were obtained, including HLA-DRB1, PTPN22, TNF, IL-6, and IL10. The gene sets of *Duhuo* active compounds and RA-related genes were imported into the Bioinformatics platform to generate a Venn diagram (see Fig. 1). Among the 273 *Duhuo*-related genes, 96 were found to overlap with the RA-related genes. The 35% overlap indicates the potential relevance of *Duhuo* in RA treatment, with representative genes including F2R, PTGS2, PDE3A, HSP90AB1, and CYP1A2.

### 3.3. Visualization of drug–disease–target interactions

**Figure 2.**
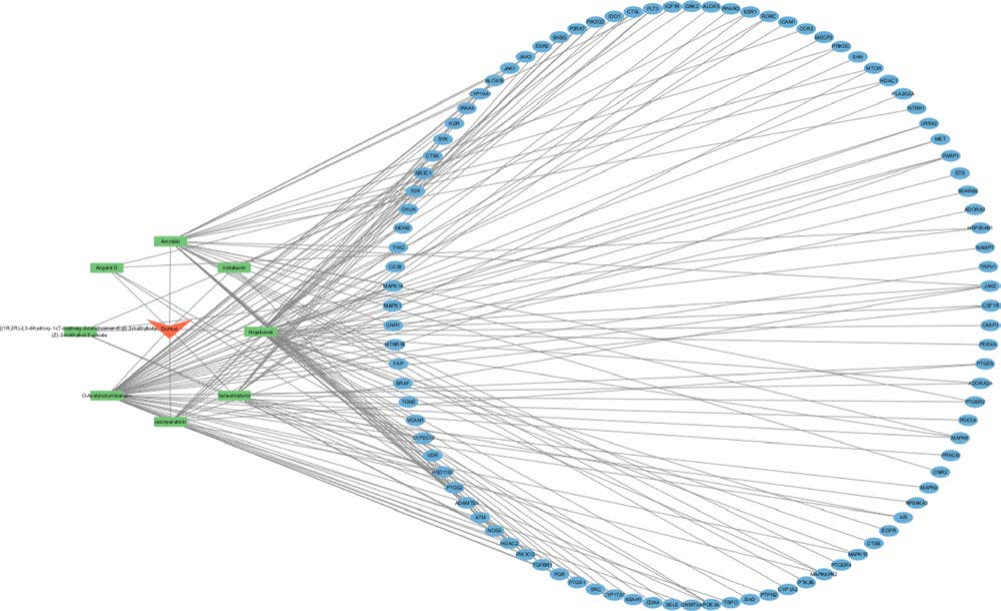
Diagram depicting Duhuo’s primary network of active ingredients, targets, drugs, and diseases. The aggregated data revealed that Duhuo’s anti-RA properties were primarily due to the presence of O-acetylcolumbianetin, angelicone, ammidin, isoimperatorin, beta-sitosterol, among others. Collaboratively addressed the objectives of PIK3CG, PTGS2, JAK2, PARP1, PIK3CD, and F2R. RA = rheumatoid arthritis.

The core genes associated with *Duhuo* and the overlapping genes with RA were imported into Cytoscape v3.10.0 to construct an interaction network depicting the relationships among the drug, active components, target genes, and disease (see Fig. 2). The network analysis indicated that *Duhuo*’s therapeutic effects against RA are mainly attributed to compounds such as O-acetylcolumbianetin, angelicone, ammidin, isoimperatorin, and β-sitosterol. These compounds were predicted to act on key targets, including PIK3CG, PTGS2, JAK2, PARP1, PIK3CD, and F2R.

### 3.4. Construction and analysis of the PPI network of *Duhuo* in RA

**Figure 3.**
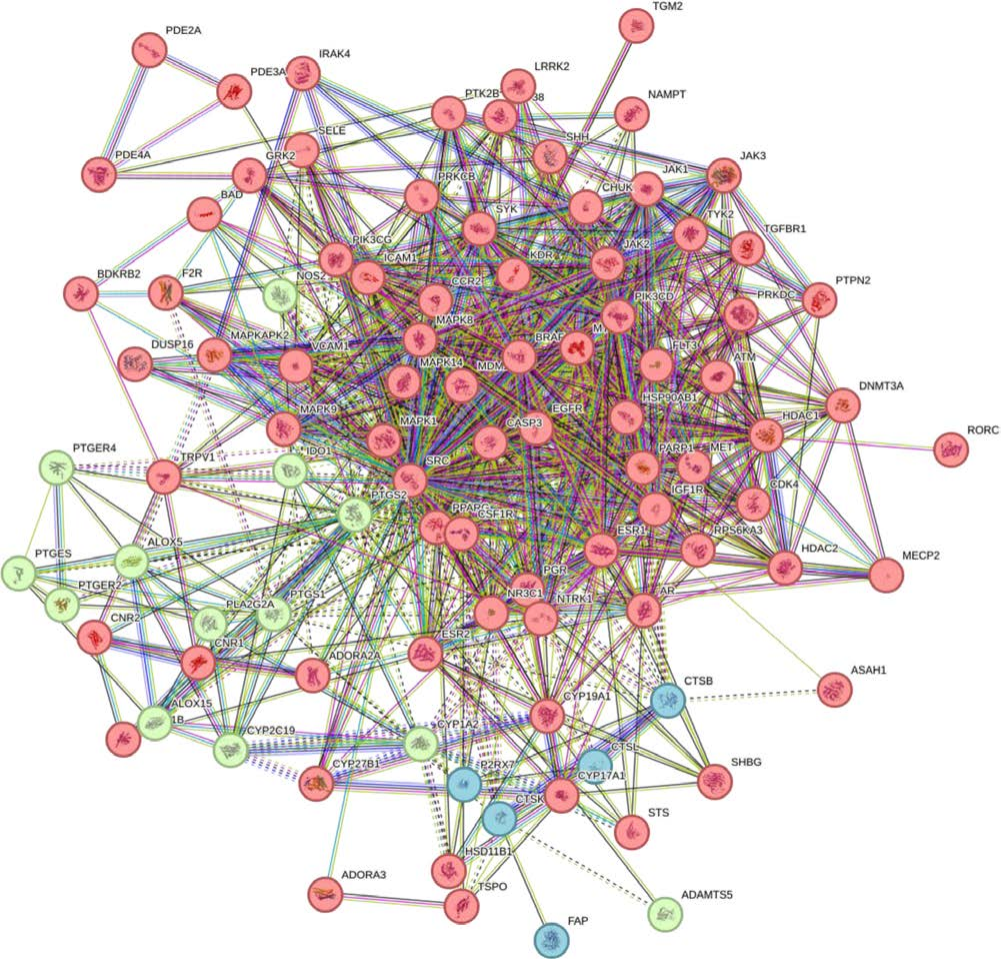
Network diagram of common target PPIs of Duhuo and RA. The interconnecting lines among nodes signify an interaction link between them, with varying colors indicating distinct interaction forms. A greater number of connecting lines indicates tighter interaction, 96 nodes, 844 edges, an average node degree of 17.6, and an average local clustering coefficient of 0.568. PPI = protein–protein interaction, RA = rheumatoid arthritis.

**Figure 4.**
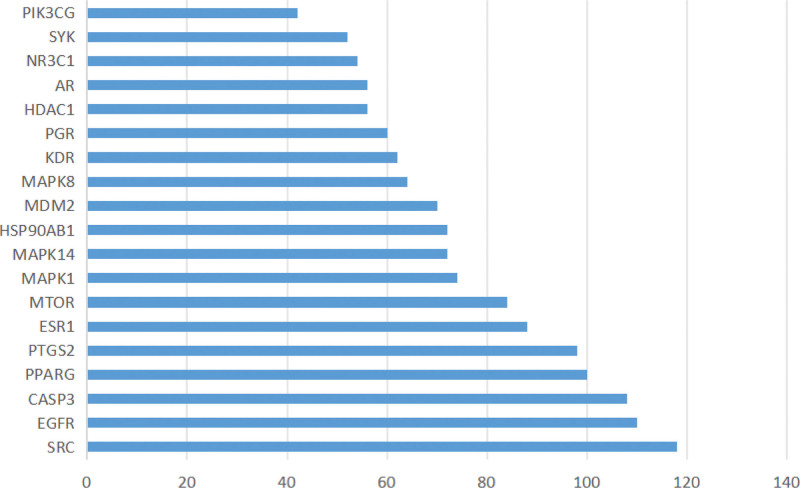
Core targets for the treatment of RA by *Duhuo*. RA = rheumatoid arthritis.

The 96 overlapping targets between *Duhuo* and RA were submitted to the STRING database with a minimum confidence score threshold of 0.4 to generate the protein–protein interaction (PPI) network (see Fig. 3). In the PPI network diagram, edges represent interactions between proteins, and different colors denote various types of associations. The resulting network contained 96 nodes and 844 edges, with an average node degree of 17.6 and a local clustering coefficient of 0.568, indicating a highly interconnected system. Cytoscape software was subsequently used to analyze the PPI data, and the top 20 hub genes were identified based on degree values. A bar plot was generated to visualize these core targets (see Fig. 4), highlighting the pivotal roles of SRC, EGFR, CASP3, PPARG, PTGS2, and other key genes within the network.

### 3.5. GO and KEGG enrichment analyses

**Figure 5.**
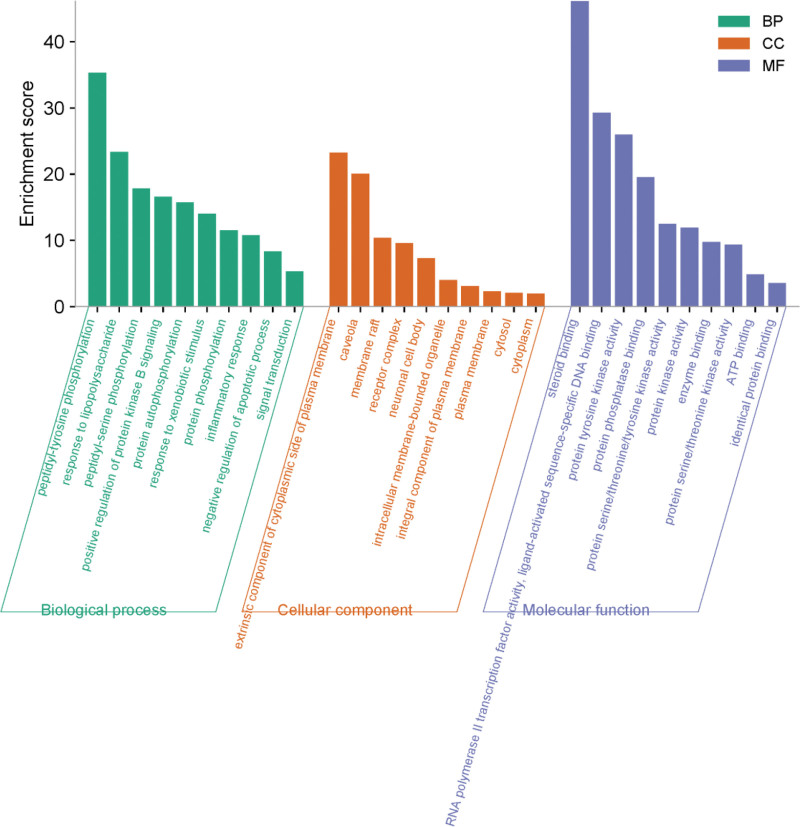
Duhuo’s triple enrichment study on BP, CC, and MF in treating RA. BP enrichment is mainly involved in various biological activities such as peptidyl-tyrosine phosphorylation, lipopolysaccharide response, peptidyl-serine phosphorylation, enhancement of protein kinase B signaling, and protein autophosphorylation. CC enrichment is primarily localized to the external part of the cytoplasmic side of the plasma membrane, including cellular components such as caveolae, membrane rafts, receptor complexes, and neuronal cell bodies on the cytoplasmic side of the plasma membrane. MF enrichment is mainly involved in molecular functions such as steroid binding, RNA polymerase II transcription factor activity, ligand-activated sequence-specific DNA binding, protein tyrosine kinase activity, RNA polymerase II transcription factor activity, protein tyrosine kinase activity, protein phosphatase binding, and protein serine/threonine/tyrosine kinase activity. BP = biological processes, CC = cellular components, MF = molecular functions, RA = rheumatoid arthritis.

The overlapping genes between *Duhuo* and RA were imported into the DAVID database, with *Homo sapiens* selected as the reference species. The top 10 enriched terms from the biological process (BP), cellular component (CC), and molecular function (MF) categories were extracted and visualized using the Microbiotics platform, as shown in Figure 5.

The BP analysis identified 468 genes mainly associated with peptidyl-tyrosine phosphorylation, response to lipopolysaccharide, peptidyl-serine phosphorylation, activation of protein kinase B signaling, protein autophosphorylation, and other essential BPs. The CC analysis involved 66 genes primarily localized to the cytoplasmic side of the plasma membrane, including structures such as caveolae, membrane rafts, receptor complexes, and neuronal cell bodies. The MF analysis revealed 104 genes mainly involved in steroid binding, RNA polymerase II transcription factor activity, ligand-activated sequence-specific DNA binding, protein tyrosine kinase activity, protein phosphatase binding, and serine/threonine/tyrosine kinase activities.

**Figure 6.**
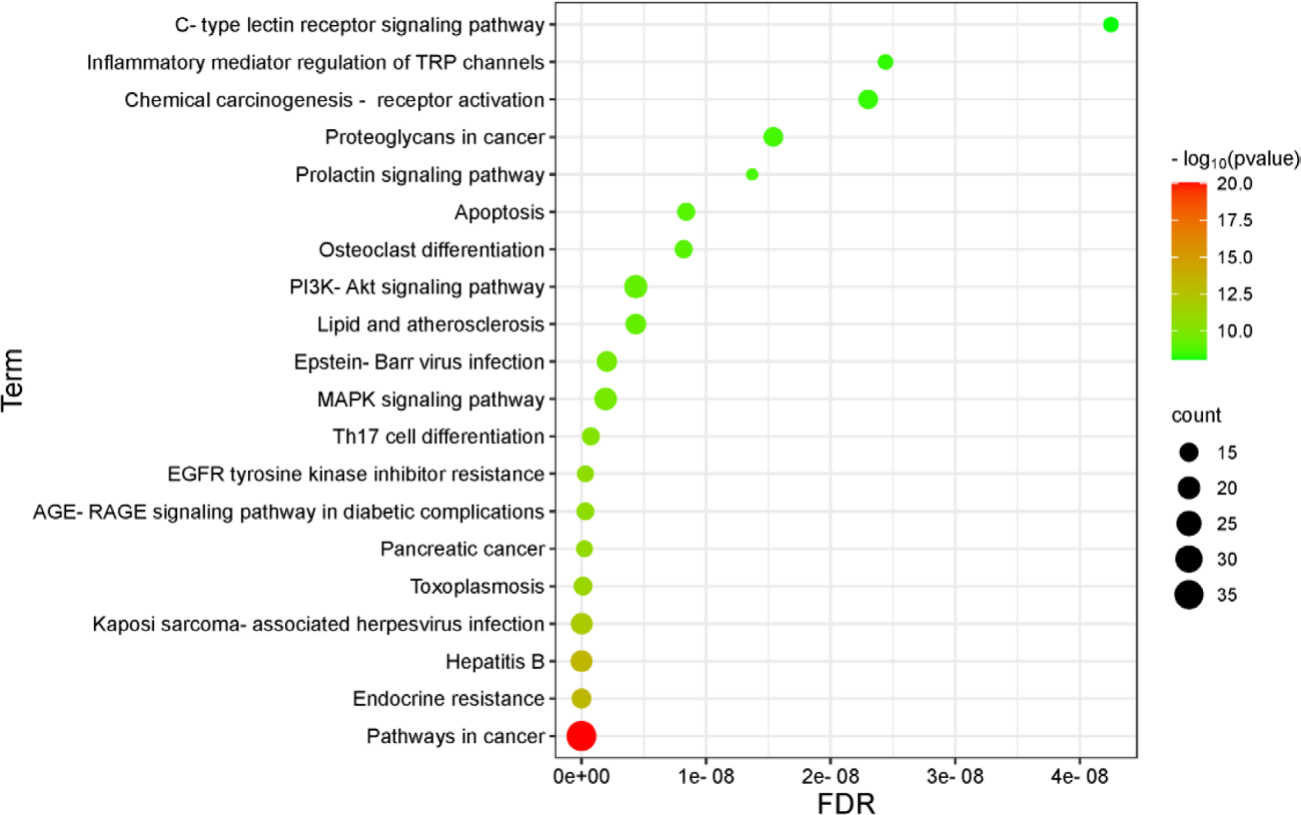
Duhuo’s KEGG pathway for treating RA. The KEGG enrichment analysis of target genes covers 152 pathways, and the top 20 pathways ranked by *P*-value are shown in the figure. The main pathways include cancer, MAPK, PI3K-Akt, prolactin, and C-type lectin receptor pathways, as well as those related to hepatitis B, endocrine resistance, toxoplasmosis, and pancreatic cancer. This indicates that the therapeutic effect of Duhuo on RA encompasses a series of biological activities, cellular components, molecular functions, and pathway mechanisms, representing a multi-dimensional biochemical process involving multiple components, targets, and pathways. KEGG = Kyoto Encyclopedia of Genes and Genomes, RA = rheumatoid arthritis.

KEGG pathway analysis identified 152 enriched pathways, with the top 20 ranked by *P*-value and visualized in Figure 6. Key pathways included those related to cancer, MAPK, PI3K-Akt, prolactin, and C-type lectin receptor signaling, as well as pathways associated with hepatitis B, endocrine resistance, toxoplasmosis, and pancreatic cancer. These findings suggest that *Duhuo* exerts therapeutic effects on RA through diverse BPs, cellular structures, MFs, and signaling pathways, reflecting its multi-component, multi-target, and multi-pathway mechanisms of action.

## 4. Discussion

RA is a chronic, systemic autoimmune disorder that primarily affects the joints but can also involve extra-articular tissues and multiple organs, including the heart, kidneys, gastrointestinal tract, and nervous system.^[21]^ Recent studies have demonstrated that the development of RA is closely associated with immune dysregulation, cytokine network imbalances, genetic susceptibility, and environmental factors, although its exact etiology remains unclear.^[22]^ Conventional western medical treatments for RA primarily include nonsteroidal anti-inflammatory drugs (NSAIDs), immunosuppressants, glucocorticoids, and biologic agents. However, these therapies are mainly symptomatic and are often accompanied by notable adverse effects.^[23]^ Therefore, the development of safe and effective alternative therapies for RA remains an urgent clinical challenge.

In recent years, TCM has attracted increasing attention in RA management due to its ability to reduce pro-inflammatory cytokine levels and alleviate pain and inflammation.^[24]^ TCM exerts therapeutic effects against RA through multi-component, multi-target, and multi-pathway mechanisms.^[25]^ *Duhuo* is a commonly used herb in RA treatment, known for its anti-inflammatory, antitumor, antioxidant, sedative, and analgesic properties.^[26]^ Although it is clinically effective, the underlying molecular mechanisms of *Duhuo* in RA treatment remain poorly understood.

In the present study, nine key active components of *Duhuo* were identified through TCMSP screening. Among them, β-sitosterol is a common dietary phytosterol found in many plant-based foods and vegetables, known for its diverse pharmacological effects.^[27]^ β-Sitosterol inhibits the VEGF signaling pathway, thereby suppressing the formation of rheumatoid synovial neovasculature,^[28]^ and exerts anti-inflammatory effects by inactivating the STAT1 and NF-κB signaling cascades.^[29]^ Animal studies have demonstrated that β-sitosterol modulates macrophage polarization by inhibiting M1-type activation, promoting M2 polarization, and effectively reducing rheumatoid inflammation.^[30]^ Isoimperatorin has been shown to suppress the mTORC1 signaling pathway, thereby delaying chondrocyte degeneration and alleviating joint inflammation.^[31]^ Furthermore, Isoimperatorin can inhibit osteoclastogenesis by blocking RANK/RANKL binding and reducing NFATc1 expression, calcium signaling, and ROS production, ultimately preventing bone resorption.^[32]^ Nodakenin and Ammidin have demonstrated notable antitumor activities. Nodakenin exerts anti-hepatocellular carcinoma effects by downregulating CXCL5 and inhibiting the ERK/MEK pathway, and also induces apoptosis and inhibits the proliferation of HeLa cells, indicating potential anticervical cancer activity.^[33]^ Ammidin promotes autophagy and has shown promising anticancer and antidrug resistance properties in cervical cancer models.^[34]^ However, there is a paucity of research on the role of these compounds in RA treatment, highlighting the need for further mechanistic studies on the pharmacological actions of other active ingredients in *Duhuo*.

Furthermore, 96 genes overlapping between *Duhuo* and RA were identified, and 20 hub targets were extracted from the STRING database. Among these, SRC, EGFR, CASP3, PPARG, and PTGS2 had the highest degree values, indicating their central roles in the PPI network. The main pathological feature of RA is the progressive degeneration of articular cartilage, which is the leading cause of joint deformity and disability in affected individuals.^[35]^ SRC, a tyrosine-protein kinase, plays a critical role in osteoclast-mediated bone resorption and inhibits osteoblast-mediated bone formation.^[36]^ In addition to regulating bone remodeling, SRC is also involved in phagocytosis, cell migration, and the production of inflammatory cytokines and mediators by macrophages – key mechanisms in the innate immune response underlying RA pathogenesis.^[37]^ EGFR signaling contributes to RA progression by promoting the proliferation of synovial fibroblasts. It is also essential for chondrogenesis during cartilage development and homeostasis, and is implicated in the aberrant expansion of synovial fibroblasts in RA.^[38]^ CASP3 plays a central role in both apoptosis and pyroptosis – two forms of programmed cell death.^[39]^ Dysregulation of these processes in synovial fibroblasts leads to their excessive accumulation, thereby exacerbating RA progression.^[9]^ PPARG is associated with the proliferation and migration of RA fibroblast-like synoviocytes (FLSs).^[40]^ It also promotes chondrocyte survival by activating the Akt/mTOR signaling pathway and inducing autophagy.^[41]^ PTGS2 (COX-2) is responsible for prostaglandin synthesis, and its reduced activity is linked to diminished inflammation and joint pain relief in RA.^[42]^ These core targets reflect the multifaceted mechanisms by which *Duhuo* may influence the course of RA through anti-inflammatory, anti-proliferative, and cartilage-protective effects.

The investigation of signaling pathways has become a central focus in modern molecular biology. Numerous studies have confirmed the pivotal regulatory roles of signaling pathways in disease development and progression. Enrichment analysis revealed that *Duhuo*’s effects on RA involve complex BPs such as peptidyl-tyrosine phosphorylation, CCs including the plasma membrane and neuronal cell body, and MFs such as steroid binding and RNA polymerase II transcription factor activity.

KEGG pathway analysis indicated that *Duhuo* is involved in multiple signaling pathways, including those related to cancer, MAPK, PI3K-Akt, and prolactin. Hallmarks of RA pathogenesis include hyperplasia of the synovial membrane and excessive secretion of pro-inflammatory cytokines.^[43]^ Evidence suggests that the aberrant proliferation of fibroblast-like synoviocytes (FLSs) in RA shares mechanistic similarities with tumorigenic pathways.^[44]^ Tumor necrosis factor (TNF) binds to its membrane receptors to mediate cell proliferation, differentiation, and apoptosis,^[45,46]^ while TNF-α promotes the expression of matrix metalloproteinases (MMPs), facilitating cartilage matrix degradation.^[47,48]^ Biologic agents commonly used in RA treatment primarily target pro-inflammatory cytokines such as TNF-α, IL-1, and IL-6, with MAPK signaling playing a central role in their regulation.^[49]^ Dysregulated MAPK signaling accelerates RA pathogenesis, and p38 MAPK has emerged as a key therapeutic target.^[50]^ In RA synovial tissue, the PI3K-Akt pathway promotes the production of cytokines such as TNF-α and VEGF,^[51]^ affecting cellular metabolism, growth, survival, and angiogenesis,^[52]^ while intensifying the inflammatory response.^[53]^ Additionally, prolactin (PRL) activates chondrocytes and synovial fibroblasts and suppresses IL-1β and IL-6 receptor expression via STAT3 phosphorylation, thereby inhibiting cartilage destruction, synovial inflammation, and osteoclastogenesis in arthritis.^[54]^

Nevertheless, this study is based solely on database analysis and network pharmacology predictions, without experimental or clinical validation. Further in vitro, in vivo, and clinical studies are necessary to confirm the pharmacological effects and mechanisms of *Duhuo* in RA.

## 5. Conclusion

Using network pharmacology, this study elucidated the potential mechanisms by which the TCM *Duhuo* exerts therapeutic effects on RA. The findings revealed that key bioactive components of *Duhuo*, such as Angelicone and O-Acetylcolumbianetin, target multiple signaling pathways and exert synergistic effects on RA pathogenesis. These compounds regulate core genes, including PTGS2 and CASP3, which are associated with immune imbalance, inflammation suppression, and inhibition of synovial cell proliferation. These interactions constitute a complex signaling network that collectively modulates RA progression.

These findings provide a scientific rationale for the use of *Duhuo* in RA treatment and underscore its advantages in multi-target, multi-system modulation. Compared with conventional Western therapies, *Duhuo* intervenes at multiple pathological levels via integrated network regulation, offering new therapeutic avenues for RA. Although this study remains theoretical, the multi-component synergistic characteristics of *Duhuo* suggest considerable therapeutic potential for RA. Further basic and clinical studies are warranted to validate these findings and facilitate the clinical application of *Duhuo*.

## Acknowledgments

We are grateful to TCMSP, PubChem, GeneCards, DisGeNET, STRING, DAVID databases, and the SwissTargetPrediction online platform for information services, the Cytoscape 3.10.0 software the Microbiology Letter platform for mapping services, and deepl (https://www.deepl.com/zh/translator) for specialized language services.

## Author contributions

**Conceptualization:** Shouyu Cao.

**Data curation:** Hongbo Li.

**Formal analysis:** Hui Lv.

**Methodology:** Shouyu Cao, Hui Lv.

**Resources:** Zilong Zhang.

**Software:** Haorui Shi.

**Visualization:** Shouyu Cao.

**Writing – original draft:** Shouyu Cao.

**Writing – review & editing:** Hongbo Li.
